# Diagnostic accuracy of low-dose dual-source cardiac computed tomography as compared to surgery in univentricular heart patients

**DOI:** 10.1186/s13019-018-0729-2

**Published:** 2018-05-16

**Authors:** Narumol Chaosuwannakit, Pattarapong Makarawate

**Affiliations:** 1 Radiology Department, Faculty of Medicine, Khon Kaen University, Khon Kaen, 40000 Thailand; 2 Cardiology Unit, Internal Medicine Department, Faculty of Medicine, Khon Kaen University, Khon Kaen, Thailand

**Keywords:** Univentricular heart, Single ventricle congenital heart disease, Cardiac CT, Dual-source CT

## Abstract

**Background:**

To evaluate the ability of low radiation dose dual-source computed tomography (DSCT) to depict the features of morphological univentricular heart and to define accuracy by comparing findings with surgery.

**Methods:**

Low radiation dose dual-source cardiac computed tomography (CCT) of 33 cases of functional univentricular heart preliminary diagnosis by echocardiography compared with the results of surgery were retrospectively analyzed (aged 1 day to 4 years, median 5 months). The appropriate dose reduction strategies and iterative reconstruction were applied.

**Results:**

Thirty three univentricular heart patients were classified into three types according to Anderson’s classification method, including 16 cases (48.5%) univentricular of right ventricular type with rudimentary chamber of left ventricle, 11 cases (33.3%) univentricular of left ventricular type with rudimentary chamber of right ventricle and 6 cases (18.2%) univentricular heart of indeterminate type without rudimentary chamber. The extracardiac malformation such as hypoplastic aortic arch, coronary artery fistula, total anomalous pulmonary venous returns or hypoplastic lung were presented frequently. The overall sensitivity and specification of cardiac CT was 100% compared to the results of surgery. The procedural dose-length product was 18 ± 5 mGy-cm, and unadjusted and adjusted radiation doses were 0.25 and 0.64 mSv, respectively.

**Conclusion:**

Cardiac CT can diagnose accurately and be performed with a low radiation exposure in patients with the functional univentricular heart disease. The aorta, pulmonary artery and lung can be evaluated completely and simultaneously as well. Cardiac CT is an effective advanced non-invasive imaging modality to comprehensive evaluation the functional univentricular heart patients, particularly if cardiac MRI poses a high risk or is contraindicated.

## Background

Functional univentricular heart disease is a spectrum of severe congenital heart disease, with multiple anatomic variations but similar surgical treatment strategies. V Functional univentricular heart disease is anatomically defined as (1) connection of both atria to the same ventricle (2:1 connection) or as (2) connection of both to atria separate ventricles, one of which is hypoplastic (1:1 connection) [1, 2]. With the advent of advanced palliative and corrective surgical procedures, Functional univentricular heart disease patients are living longer into adulthood compared to two or three decades ago [3], and they are more frequently undergoing imaging to assist in clinical and surgical management. However, interpreting an imaging examination of a functional univentricular heart disease patient is a daunting task, not only because of unusual anatomy and varied post operative appearances but also because of the rarity of the functional univentricular heart disease that makes it difficult for the imaging specialist to maintain diagnostic proficiency.

Echocardiography and cardiac magnetic resonance imaging (CMR) are the main imaging modalities used in adult patients with complex congenital heart disease. However, cardiac CT offers complementary imaging and can be performed safely in unwell patients with single ventricle physiology. Echocardiography is insufficient for evaluation of the thoracic vasculature or for reproducible estimation of ventricular function [4, 5]. Cardiac MRI (CMR) is commonly performed for these indications, but it requires relatively long imaging times, deep sedation, or anesthesia in young children. Many older patients have metallic implants with an artifact that degrades magnetic resonance imaging (MRI) quality [6]. In addition, it is relatively contraindicated in those with pacemakers and defibrillators as these devices have been known to cause an imaging artifact [7]. Recent findings of cerebral gadolinium deposits suggest that MRI use be carefully considered when angiography is needed [8–10]. Cardiac CT has been shown to be accurate for the evaluation of anatomy and function for most indications of congenital heart disease (CHD), [11] but there has not been a report on image quality, nor has a correlation been made with interventional findings, in a cohort of single-ventricle patients across all stages of palliation.

## Methods

### Patient population

This retrospective study included 33 functional univentricular heart disease patients who underwent both cardiac CT and surgery, between February 2013–December 2016. The indications for cardiac CT were pre-operative evaluation of complex congenital heart and functional univentricular heart disease patients which is considered appropriate indications for cardiac CT, based on the expert consensus document of the Society of Cardiovascular computed tomography [11]. Exclusion criteria for cardiac CT included the presence of renal failure, and a history of allergic reaction to iodine-containing contrast agents. The present study was approved by the Ethics Committee of the Faculty of Medicine, Khon Kaen University, Khon Kaen, Thailand, and informed consent was obtained from all patients The IRB protocol number of this study is 800001189.

### Dual-source cardiac CT (CCT) scanning protocol

No sedation versus sedation with free breathing versus intubation, IV site and gauge, and adverse procedural events were determined from patient records. Nearly all patients could be performed cardiac CT with limited or no anaesthesia and with quiet respiration. Imaging was performed by using a second-generation dual-source CT scanner (Somatom Definition; Siemens Healthcare, Forchheim, Germany) with temporal resolution = 75 ms. The radiation dose is kept to minimum by reducing the kilovoltage and tube current appropriately. For children weighing less than 10 kg, 10–19 kg and 20–30 kg, we use 80 kV and 80 mAs, 80 kV and 100 mAs, and 100 kV and 120 mAs, respectively. Other cardiac CT parameters were as follows: number of X-ray tubes, two; collimation, 128 detector rows of 0.5 mm each, with double sampling by using rapid alteration of the focal spot in the longitudinal direction (*z* flying focal spot). Prior to scanning, the pitch was set automatically by the scanner software. Depending on heart rate, pitch was set between 0.2 and 0.43. Automated dose regulation methods such as CARE dose 4D (Siemens Healthcare) may be used to reduce the radiation. A bolus of iodinated contrast material (350 mg/mL, Omnipaque; GE Healthcare) at a dose of 1.5 ml/kg with dual-head power injector at a rate of 1.5–2.0 ml/s for a 22-gauge cannula, 3.0 ml/s for a 20- gauge cannula and 4.0–5.0 ml/s for a 18-gauge cannula followed by a 10–20 ml of saline flush at a same rate to that of the contrast injection. For timing purposes, an automated bolus-tracking software was used, starting the scan automatically 6 s after contrast agent density in the descending aorta reached a predefined threshold of 130 HU. The entire volume of the heart and pulmonary arteries was covered during one breath-hold in approximately 5 s with simultaneous recording of the ECG trace. Patients were scanned in the supine position. Cardiac CT is performed from the thoracic inlet level to L1–L2. When there is suspicion of anomalous pulmonary venous drainage, the scan can be extended down to the lower border of the liver.

### Cardiac CT image analysis

Cardiac CT image analysis was performed by two cardiovascular and thoracic radiologists in consensus (with a respective 11 and 10 years of experience in examining cardiovascular and thoracic CT scans) and blinded to the clinical data and the results of surgery. First, axial all image data are evaluated using a 3D post processing workstation with Syngo software (Siemens Healthcare). Various image reformatting techniques including curved planar reconstruction, maximum intensity projection (MIP), minimum intensity projection, and volume-rendering technique (VRT) are used to get all the clinically relevant information. Curved planar reformatting and MIP are primarily used to evaluate curved structures such as the pulmonary arteries and major aortopulmonary collateral arteries (MAPCAs). Minimum intensity projection is used to evaluate the airway and lung parenchyma. For 3D reformatting of the complex anatomy, VRT is used. Thin-section multiplanar reformatting is used for quantitative analysis of the structure in question.

### Cardiac surgery result analysis

Cardiac surgery result analysis was performed by an experienced cardiologist blinded to cardiac CT results by retrospectively reviewed operative notes.

### Radiation dose parameters

The scanner platform, contrast, imaging sequence, CT dose–volume index, milligray (mGy), scan dose–length product (mGy-cm, 16 cm phantom), scan length, tube potential, and tube current were recorded for each scan. Individual scan and cumulative procedural dose–length products in mGy-cm were recorded.

### Radiation dose estimation

Procedural dose–length product was used to estimate the radiation dose. An unadjusted radiation dose in milliSievert (mSv) was calculated by multiplying the dose– length product with the standard chest conversion factor given as scan dose–length product × 0.014 [12]. For patients < 18 years of age, conversion factors were further calculated by age as follows: 0.039 for ≤0.50 years; 0.026 for 0.51–2.50 years; 0.018 for 2.51–7.50 years; and 0.014 for patients > 7.50 years [13, 14].

### Statistical analysis

Continuous data were expressed as mean ± SD. Statistical analyses were performed using SPSS software version 16 (SPSS, Inc., Chicago, IL, USA). A significance level of *p* < 0.05 was considered a statistically significant result and all reported *p*-values were two-sided. Means were compared using unpaired t-test, and Mann-Whitney rank sum was used when data was not normally distributed.

## Results

Thirty-three patients (20 boys, 13 girls; aged 1 day to 4 years, median 5 months) were included in this study over a period of 44 months. The mean time interval between cardiac CT and surgery was 1 ± 10 months (range, 3 days–14 months) and there were no clinical events between the 2 studies in any patient. Patient demographic data and cardiac CT scan information are lists as Table 1.

**Table 1:** Patient demographic data and cardiac CT information

Characteristic	Value
Age at scan (months), mean ± SD (range)	5 ± 8 (0–48)
Men, *n* (%)	20 (60.6%)
Height (cm), mean ± SD	90.8 ± 23
Weight (kg), mean ± SD	3 ± 10
Patient sedation	
• No sedation, *n* (%)	10 (30.3)
• Sedation with free breathing, *n* (%)	22 (66.6)
• Intubation, *n* (%)	1 (3.1)
Dose-length product (mGy-cm), mean ± SD (range)	18 ± 5 (15–21)
Unadjusted radiation dose (milliSievert), mean (range)	0.25 (0.21–0.29)
Adjusted radiation dose (milliSievert), mean (range)	0.64 (0.49–0.82)

**Fig. 1:**
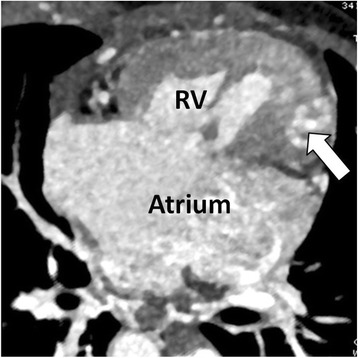
Cardiac CT of a 2 days old infant showing single atrium (Atrium), univentricular right ventricle (RV) and rudimentary left ventricle (arrow)

**Fig. 2:**
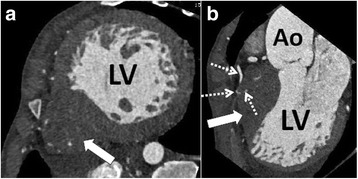
Cardiac CT of a 1 month old boy showing univentricular left ventricle (LV) and rudimentary right ventricle (**a** and **b**; arrows). Coronary artery fistula from right coronary artery draining into the rudimentary right ventricle was found (**b**; dashed arrows)

In patient analysis, out of 33 cardiac CT examinations, none of them was of non-diagnostic image quality. Thirty three univentricular heart patients were classified into three types according to Anderson’s classification method, including 16 cases (48.5%) univentricular of right ventricular type with rudimentary chamber of left ventricle (Fig. 1), 11 cases (33.3%) univentricular of left ventricular type with rudimentary chamber of right ventricle (Fig. 2) and 6 cases (18.2%) univentricular heart of indeterminate type without rudimentary chamber.

**Fig. 3:**
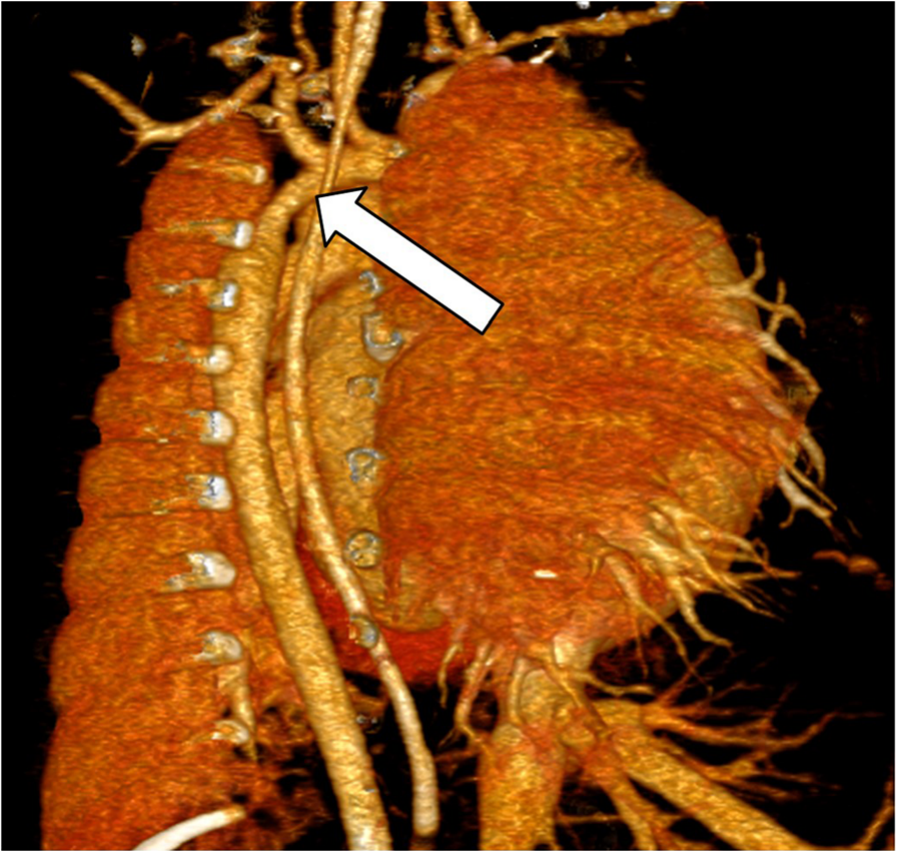
Cardiac CT of a 1 month old univentricular right ventricle patient showing hypoplastic aortic arch (arrow)

**Fig. 4:**
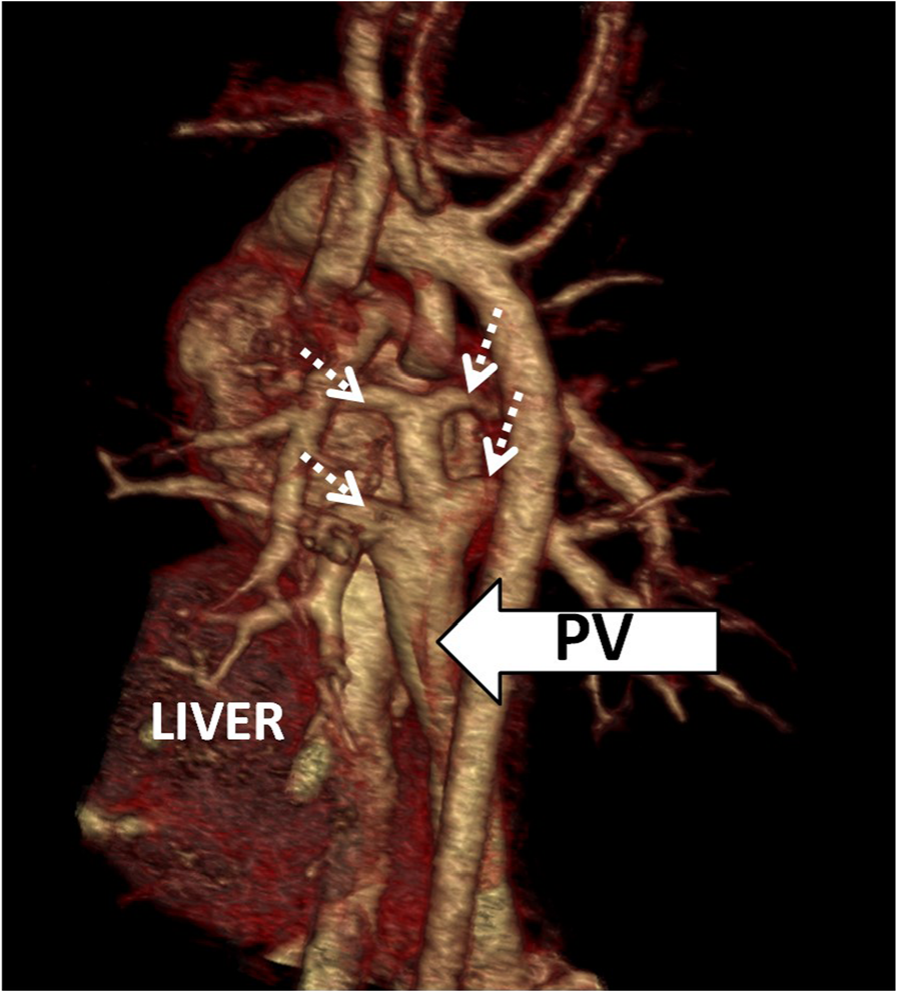
Cardiac CT of a 1 year old univentricular left ventricle patient showing infracardiac type total anomalous pulmonary venous returns (dashed arrows) which draining into portal vein (arrow). (PV; portal vein)

Of the 33 patients, significant unexpected findings were two hypoplastic aortic arch (Fig. 3), three coronary artery fistula (Fig. 2), ten pulmonary atresia, one infracardiac type total anomalous pulmonary venous returns (Fig. 4) and one hypoplastic left lung were presented. Ten patients were scanned without sedation, 22 patients were scanned with minimal to moderate sedation, and one patient was intubated during the scan. The patient who was intubated for general anesthesia for multiple concurrent procedures including brain CT and cardiac CT.

The procedural dose-length product was 18 ± 5 (15–21) mGy-cm, and unadjusted and adjusted radiation doses were 0.25 (0.21–0.29) and 0.64 (0.49–0.82) mSv, respectively.

All patients underwent subsequent staged palliation. No patient had additional advanced diagnostic studies before initial surgical palliation. No discrepancy was found of opinion regarding the classification of univentricular heart disease at time of surgery compared to cardiac CT findings. The overall sensitivity and specification of CTA was 100% compared to the results of surgery.

## Discussion

CT is a robust alternative diagnostic modality for diagnosis of functional univentricular heart disease. In a significant number of patients in our cohort who had previous echocardiography, cardiac CT was able to provide additional information on extracardiac findings such as hypoplastic aortic arch, infracardiac type total anomalous pulmonary venous returns and hypoplastic left lung. For highly select indications, the risk profile may sometimes be in favour of using cardiac CT compared with other diagnostic methods when risks from anaesthesia are considered. Univentricular heart patients are exposed to relatively high cumulative radiation levels during staged palliation [15, 16]. A single institution reports a median cumulative effective radiation dose of 25.7 mSv from birth to 33 months of age, of which 78% was from catheterization [15]. Another study of cumulative radiation dose for patients with all forms of congenital heart disease showed that 5.3% of patients received over 20 mSv/year with a median follow-up time of 4.3 years [17].

A recent study directly comparing radiation doses from diagnostic catheterization (*n* = 50 cases) and computed tomography angiography (n = 50 cases) in children with congenital heart disease has shown 15-fold less radiation from CT angiography, although this was not specific to patients with single-ventricle heart disease [18]. Other studies using older CT scanner showing doses for CT angiography (*n* = 21) that are twofold higher than those in diagnostic cardiac catheterization (*n* = 117) [18]. These results show that the radiation dose from CT varies considerably depending on the type of scanner used and the aggressiveness of dose reduction. The image quality necessary for evaluation of coronary lesions in adult patients is rarely required for congenital applications, and patient-specific dose reduction must be implemented if a diagnostic strategy utilising CT is to be implemented. The standard diagnostic protocol at a majority of centers remains invasive catheterization before surgical palliation, despite data showing a favorable risk profile for non-invasive evaluation [3]. Non-invasive assessment before surgical palliation has shown similar operative outcomes compared with invasive catheterization involving lower risk as measured by radiation exposure, vascular access complications, length of anesthesia, and adverse events [19–21].

Our practice now uses CT preferentially for evaluation of anatomy before surgical palliation. Catheterization is preserved for patients in whom intervention is likely considered on the basis of echocardiography or clinical examination, and for patients with poor ventricular function and severe valve regurgitation in whom hemodynamics are considered relevant to clinical management. Some experts now propose a non-invasive algorythm for evaluation before surgical palliation in patients with single-ventricle heart disease considered to be at a low risk for requiring intervention [22–24].

Cardiovascular MRI is the most commonly used non-invasive advanced imaging modality in congenital heart disease but deep sedation or general anesthesia is required in young children, scan times are relatively long, and gadolinium is used in many patients for angiography. Anesthesia poses increased risk for patients with complex congenital heart disease undergoing MRI evaluation, and there is concern that repeated anesthesia exposure of young patients may have adverse neurological effects [25–34]. Gadolinium deposits have been found in brain tissue after repeated dosing in both children and adult patients, the significance of which is not yet known [35–38]. Risk assessment of non-invasive modalities should include assessment of risk from anesthesia and iodinated or gadolinium-based contrast exposure in addition to radiation exposure and vascular-access requirements.

Cardiac CT is an alternative imaging modality that can be used as part of the non-invasive imaging modality when cardiac MRI is considered to pose a high risk or when there is an imaging artifact [39, 40]. When anesthesia is needed, a single breath-hold is required for data acquisition and the length of anesthesia will be relatively short.

Despite promising initial results, our study has potential limitations. The findings from the present study are retrospective and limited to single-center experience, the generalizability of the present results is limited. The present result is also limited by the number of patients, therefore, the interpretation of sensitivity may be limited.

## Conclusion

Cardiac CT can diagnose accurately and be performed with a low radiation exposure in patients with the functional univentricular heart disease. The aorta, pulmonary artery and lung can be evaluated completely and simultaneously as well. Cardiac CT is an effective advanced non-invasive imaging modality to comprehensive evaluation the functional univentricular heart patients, particularly if cardiac MRI poses a high risk or is contraindicated.

## Abbreviations

CCT: Cardiac computed tomography
CHD: Congenital heart disease
CMR: Cardiac magnetic resonance imaging
CT: Computed tomography
MAPCAs: Major aortopulmonary collateral arteries
mGy: Milligray
MIP: Maximum intensity projection
MRI: Magnetic resonance imaging
mSv: Millisievert
VRT: Volume-rendering technique
